# Dental Implant Displacement Into the Maxillary Sinus Ostium: A Case Report of Customized Surgical Management

**DOI:** 10.7759/cureus.106421

**Published:** 2026-04-04

**Authors:** Metin Berk Kasapoglu, Alanur Sahabettinoglu, Belir Atalay

**Affiliations:** 1 Department of Oral and Maxillofacial Surgery, Faculty of Dentistry, Istanbul University, Istanbul, TUR

**Keywords:** cone-beam computed tomography, customized surgical instrument, dental implant displacement, intraoral retrieval, maxillary sinus

## Abstract

Displacement of dental implants into the maxillary sinus is an uncommon yet clinically significant complication in implant dentistry. Migration toward the maxillary sinus ostium is rare and presents unique management challenges. This case report describes a 75-year-old woman who experienced intraoperative implant displacement into the left maxillary sinus during full-arch rehabilitation. Initially positioned at the sinus floor, the implant was later found at the ostium on cone-beam computed tomography one week after the incident, likely due to patient head movement. The patient reported mild discomfort but showed no signs of sinusitis or oroantral communication. Declining endoscopic surgery, she opted for a less invasive intraoral approach under local anesthesia. A custom-modified dental explorer with a notched tip was designed to securely engage the implant threads. A lateral sinus window was created, and indirect visualization was achieved using rhodium-coated mirrors. The implant was successfully retrieved through the intraoral route, and the sinus entry was sealed using a Bichat fat pad graft. Postoperative management included antibiotics, nasal decongestants, and analgesics. Healing progressed uneventfully, with no complications observed at 10-day, one-month, and three-month follow-ups. This case underscores the potential for dynamic implant migration within the sinus and demonstrates the effectiveness of customized instrumentation and early intervention in achieving successful, minimally invasive retrieval without the need for endoscopic techniques.

## Introduction

Dental implant displacement into the maxillary sinus is a rare but clinically significant complication in implant dentistry, with the available evidence largely limited to case reports and small case series [1]. The maxillary sinus is a pyramidal cavity within the maxilla and is lined by the Schneiderian membrane, a ciliated respiratory mucosa responsible for mucociliary clearance toward the natural ostium. In the posterior maxilla, sinus pneumatization, reduced residual bone height, and poor bone quality may increase the risk of implant displacement into the sinus during or after implant placement [2]. Although mucociliary activity plays an essential role in sinus homeostasis, secondary positional change of a displaced implant is more likely influenced by mechanical factors such as gravity, repetitive head movements, sinus anatomy, and implant-related characteristics [3,4].

Several techniques have been described in the literature for implant removal, such as the Caldwell-Luc procedure, which creates a window in the anterior sinus wall, the bone lid technique, and the lateral antrostomy technique, or its modifications, are commonly utilized [3,4]. In recent years, endoscopic approaches, including transnasal and transoral methods, have been increasingly favored for their minimal invasiveness, superior visibility, and reduced postoperative complications [5].

This report presents a rare case of implant migration within the maxillary sinus, emphasizing the dynamic displacement potential caused by excessive head movement. On the day of the incident, periapical radiography identified the implant at the sinus floor, whereas cone-beam computed tomography (CBCT) performed a week later revealed its translocation to the ostium, likely due to patient mobility [6,7]. Despite the challenges posed by this movement, the implant was successfully retrieved intraorally using indirect vision with rhodium dental mirrors and a custom-modified dental explorer. The explorer, adapted from a standard dental tool, was preshaped to securely engage the implant’s threads and extend to the ostium for effective retrieval. This case highlights the critical importance of comprehensive preoperative planning, anticipating potential implant migration routes, and using patient-specific retrieval tools for successful intraoral management of displaced implants within the maxillary sinus.

This article was previously posted as a preprint on the Authorea server on June 13, 2025.

## Case presentation

**Figure 1:**
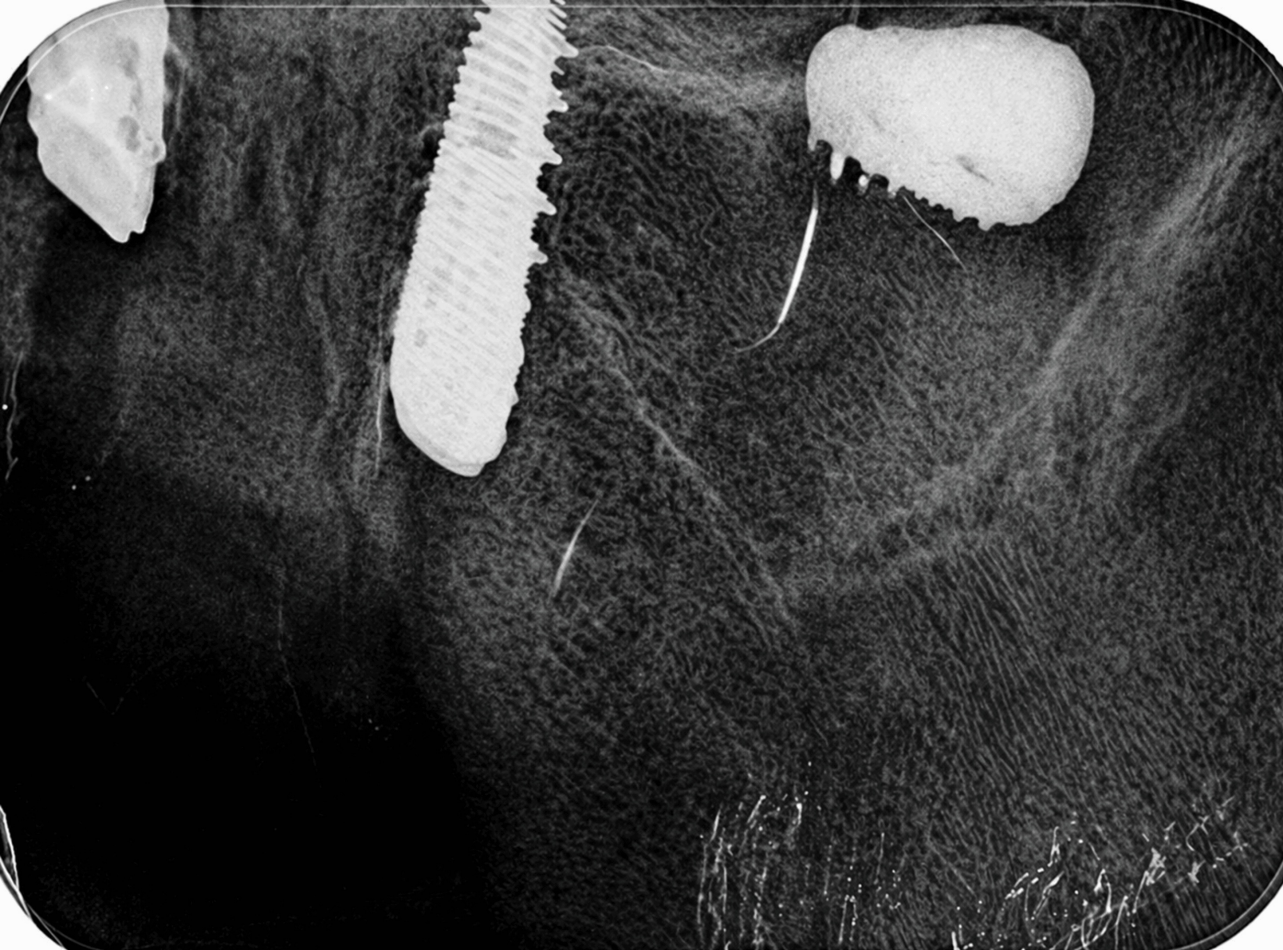
Periapical radiograph showing the dental implant displaced into the left maxillary sinus

**Figure 2:**
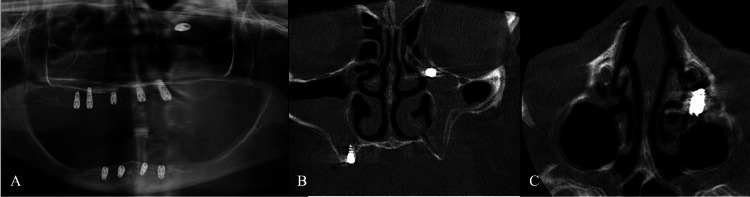
Radiological views of the displaced implant. Panoramic radiograph showing the displaced implant oriented horizontally and extending toward the orbital floor and nasal cavity (A). Coronal section from CBCT confirming proximity to the nasal cavity (B). Axial CBCT section showing horizontal orientation of the implant within the sinus (C) CBCT: cone-beam computed tomography

A 75-year-old female patient presented to a private clinic-seeking placement of a dental implant. The treatment plan comprised a fixed prosthesis for the maxilla supported by six implants and a removable prosthesis for the mandible supported by four implants. All implants were scheduled to be placed under local anesthesia within a single session. The initial implant placement was performed at this external clinic using a free-hand technique. Detailed implant specifications, including brand, diameter, and length, were unavailable because the procedure was not performed at our institution. During the placement of the final implant in the upper left region, displacement of the implant into the maxillary sinus occurred. The attending clinician attempted retrieval from the socket; however, after loss of visibility, periapical radiography was performed, revealing implant migration into the sinus (Figure 1). The clinician subsequently closed the surgical flap, prescribed amoxicillin with clavulanic acid for infection prophylaxis, and referred the patient to a specialized center for further evaluation and management. One week later, the patient presented to the Department of Oral and Maxillofacial Surgery, Faculty of Dentistry, Istanbul University. Clinical examination revealed mild pain localized to the upper left region, slight gingival edema, and tenderness on palpation, with no evidence of trismus. There were no clinical signs indicative of acute sinusitis, nasal discharge, or oroantral communication, and the mucosal integrity at the socket site remained intact. Panoramic radiographic imaging identified the implant positioned horizontally within the maxillary sinus, extending toward the orbital floor and nasal cavity (Figure 2A). To precisely determine the implant’s location, CBCT was performed, confirming its lodgment at the orifice of the left ostium (Figures 2B, 2C). Because CBCT demonstrated that the implant was lodged near the maxillary ostium, timely retrieval was considered necessary to prevent further migration and possible ostium-related complications. Although endoscopic retrieval is a well-recognized minimally invasive option, an intraoral approach was preferred in this case because it allowed direct access to the implant position with controlled surgical exposure and without the need for a transnasal intervention.

**Figure 3:**
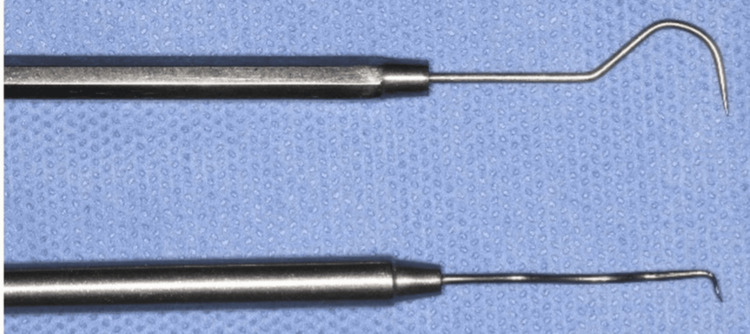
Custom-modified dental explorer with a notched tip, designed to engage the implant threads for retrieval

A standard dental explorer was modified chairside preoperatively by the surgical team into a custom-made instrument with a small notched tip, designed to securely engage the implant threads and facilitate its retrieval from the ostium (Figure 3).

**Figure 4:**
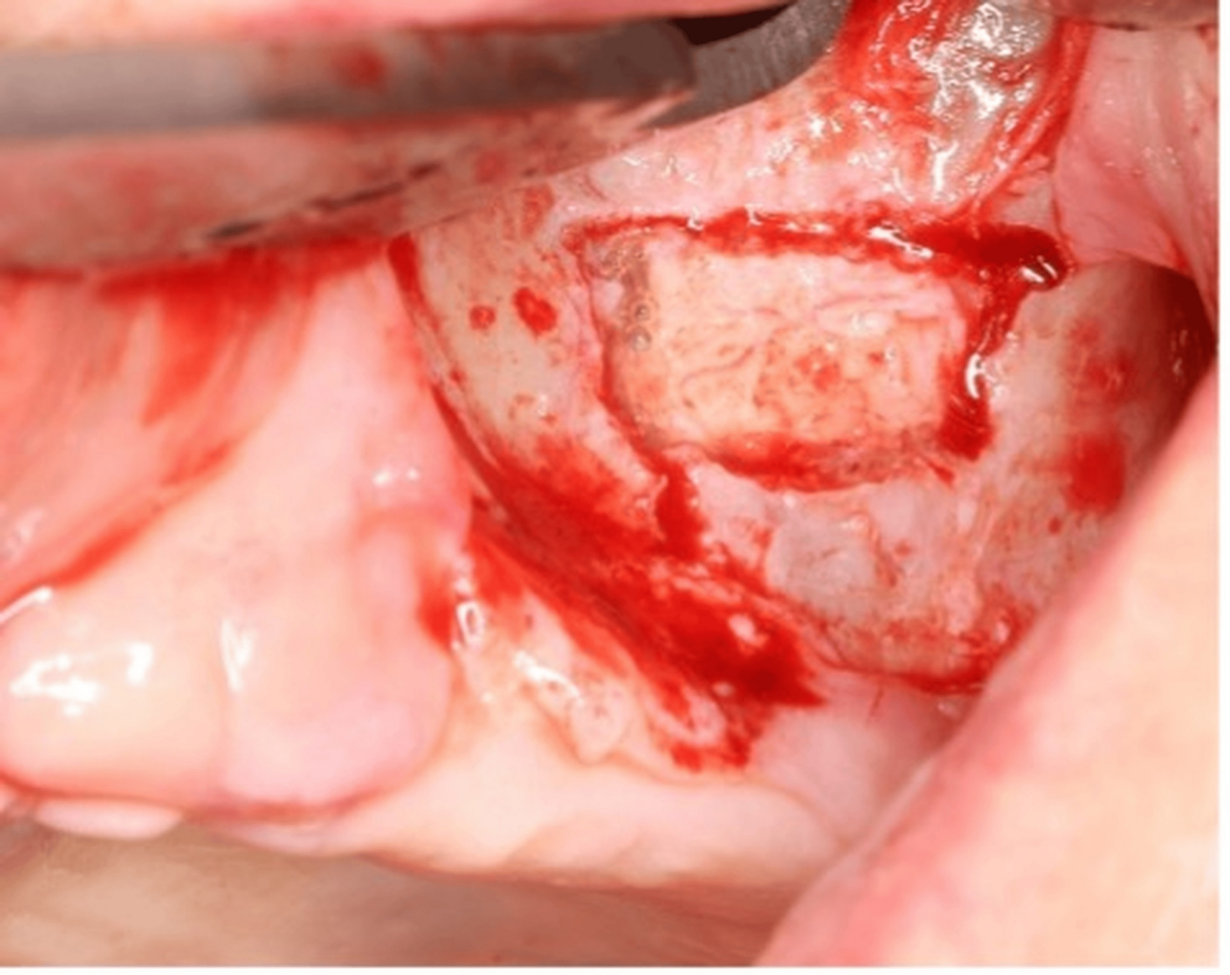
Intraoperative image showing the creation of a bony window in the lateral wall of the maxillary sinus

To minimize the risk of oroantral fistula formation and preserve alveolar crest healing at the site of implant migration, a superior buccal incision was made instead of a crestal incision. This approach aimed to maintain mucosal integrity at the site of sinus entry, optimizing postoperative healing and reducing complications. Using a surgical handpiece under copious saline irrigation, a bony window was meticulously created in the lateral sinus wall utilizing tungsten carbide burs (Figure 4).

**Figure 5:**
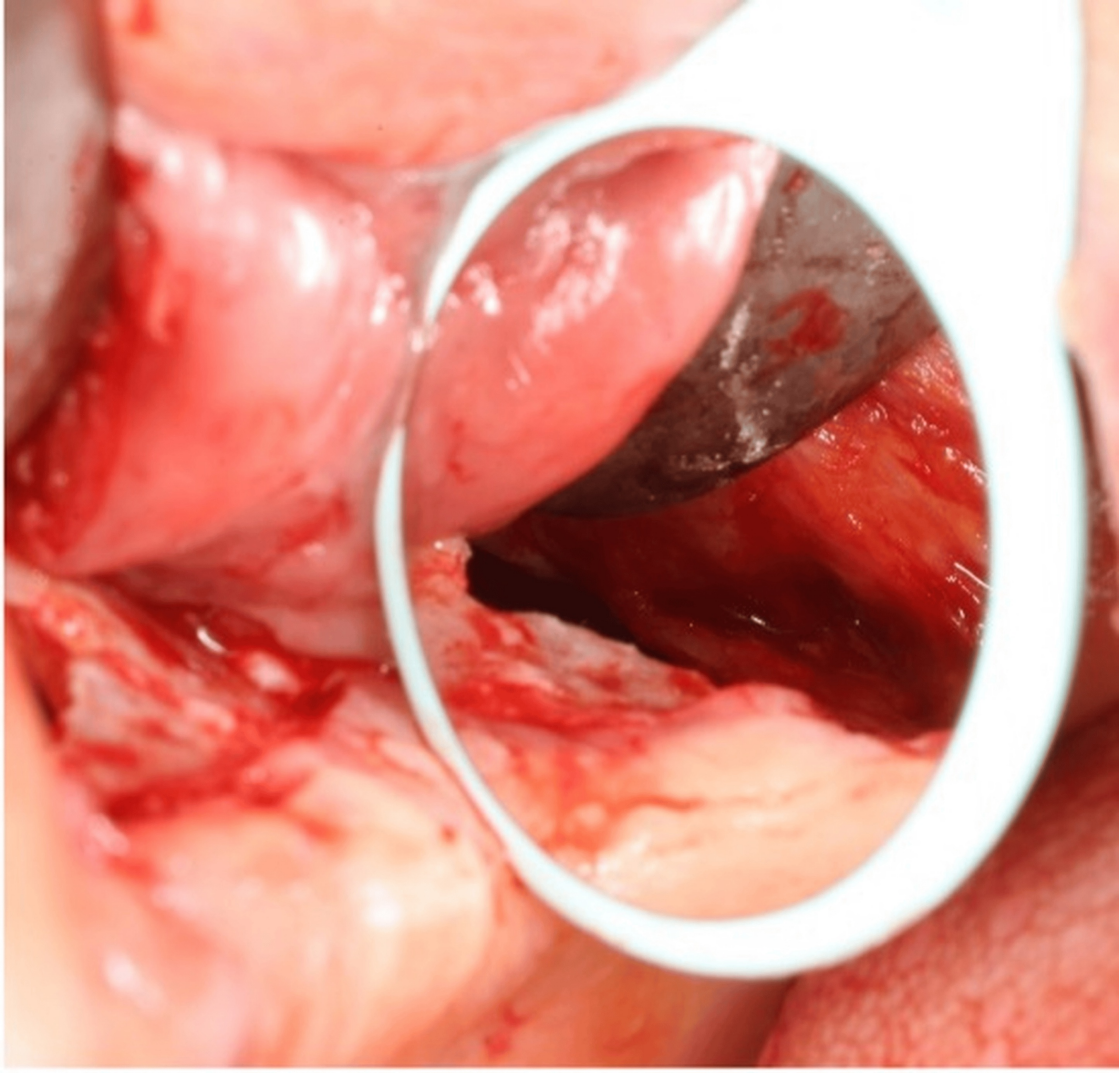
Intraoperative use of rhodium-coated dental mirror for indirect visualization of the implant within the maxillary sinus

The surrounding mucosa and granulation tissue, which had formed due to the one-week delay, were carefully incised using a #12 scalpel to ensure optimal visualization of the displaced implant within the maxillary sinus. Rhodium-coated dental mirrors, selected for their nonfogging and highly reflective properties, were employed to facilitate intraoperative monitoring of the implant’s position within the maxillary sinus (Figure 5).

**Figure 6:**
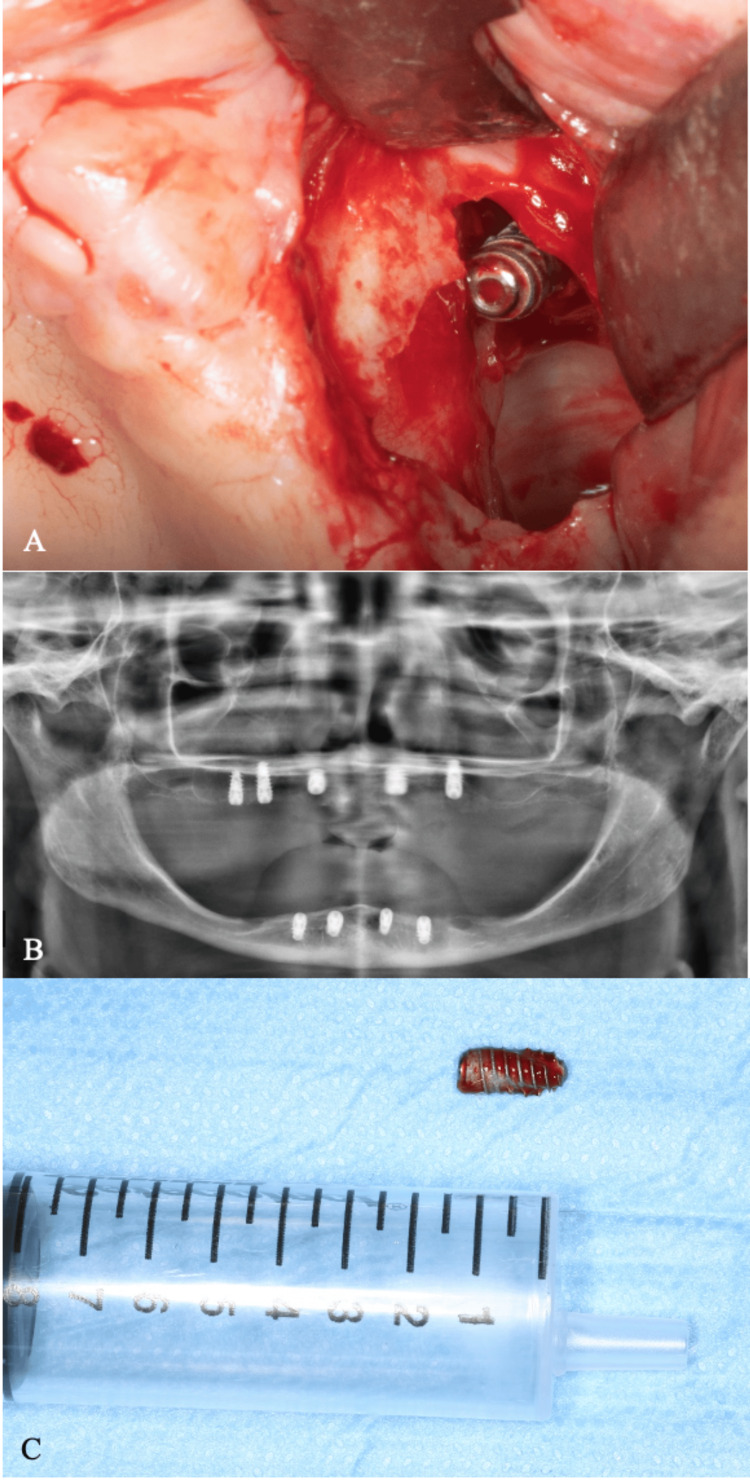
Retrieval of the implant. Intraoral view showing the implant within the maxillary sinus prior to retrieval (A). Postoperative panoramic radiograph taken after retrieval of the implant (B). Clinical photograph of the removed implant (C)

Direct visualization of the ostium often required significant surgeon tilting; therefore, indirect visualization through mirrors proved essential. Utilizing both direct and indirect visualization, the implant was securely grasped and successfully retrieved using the custom-made notched dental explorer (Figures 6A-6C).

**Figure 7:**
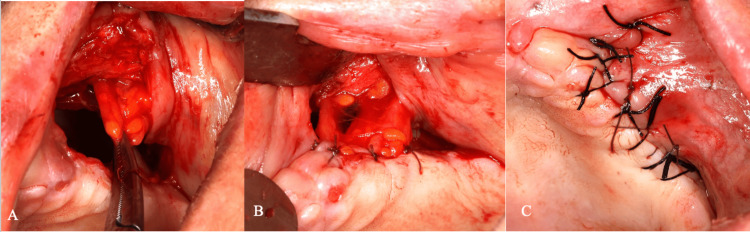
Closure of the surgical site. Placement of a Bichat fat pad graft at the surgical site (A). Mucosal closure of the bony window area (B). Final view after primary closure (C)

The bony window was subsequently closed with a Bichat fat pad (BFP) graft and covered with a primary closure flap, due to its rich vascularity, ease of mobilization, and high success rate in preventing oroantral fistula formation (Figures 7A-7C).

**Figure 8:**
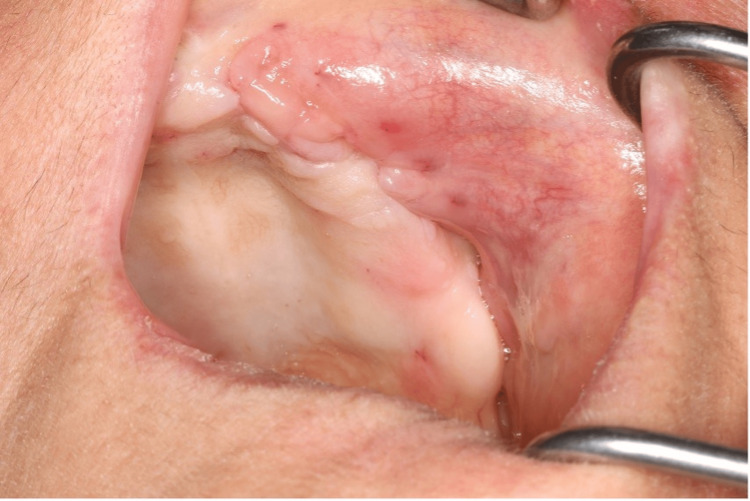
Clinical follow-up at three months demonstrating uneventful healing and intact mucosal integrity

The patient’s postoperative recovery was uneventful, with no evidence of infection or oroantral communication noted at follow-up evaluations conducted at 10 days, one month, and three months, confirming stable healing without complications (Figure 8).

## Discussion

Dental implant displacement into the maxillary sinus is an uncommon yet clinically significant complication that may occur during or after surgical placement [2,4,7]. Contributing factors include insufficient bone quality or quantity in the posterior maxilla, alveolar pneumatization, and improper surgical techniques [8]. Technical errors, such as overpreparation of the implant site, sinus floor perforation, and failure to achieve adequate primary implant stability, are often key contributors. Additionally, poor planning, surgical inexperience, and inadequate assessment of the patient’s anatomical complexities can lead to intraoperative implant displacement [9,10].

In this case, the absence of bone augmentation techniques in a patient with advanced age and challenging anatomy, combined with the decision to place 10 implants in a single session, significantly increased the risk of complications. The displacement of the final implant during the session underscores the importance of surgeon expertise, particularly during prolonged surgical procedures. Patient fatigue and the surgeon's potential loss of concentration in extended sessions likely compounded the risk.

In cases of dental implant displacement into the maxillary sinus, immediate removal is often recommended in the literature to prevent complications [11]. Granulation tissue and infection surrounding the implant can ease its movement within the sinus, further complicating the situation [12]. Consequently, early removal of a displaced implant is critical to minimizing risks of sinusitis, oroantral fistula, and other associated complications [9,10]. Pharmacologic management typically includes broad-spectrum antibiotics, analgesics, and nasal decongestants to prevent infection and support healing [11]. In the present case, postoperative management consisted of amoxicillin-clavulanic acid (875/125 mg), nonsteroidal anti-inflammatory analgesics, and nasal decongestants. This regimen was selected considering the patient’s advanced age, absence of systemic disease, and standard postoperative protocols, and no adverse drug reactions were observed.

Mucociliary activity within the maxillary sinus plays a crucial role in maintaining sinus hygiene by transporting mucus toward the ostium. However, mucociliary activity alone is considered insufficient to displace a dental implant. It has been suggested that displaced implants may undergo secondary migration within the maxillary sinus due to a combination of mechanical and physiological factors. In particular, mucociliary clearance, in conjunction with repetitive head movements, may contribute to positional changes toward the natural ostium [13]. In our case, an 8-mm implant was selected by the operating clinician, considering the limited bone volume in the region, and we believe this significantly contributed to the implant’s rapid migration to the ostium orifice within days [7,8]. Additionally, granulation tissue formation around the implant was observed and is considered a result of the delayed intervention.

Imaging techniques, including panoramic radiography, Waters’ views, and computed tomography, are essential for determining the implant’s exact location following its migration into the sinus. In our case, an initial periapical radiograph showed the implant at the sinus floor, while a panoramic radiograph taken one week later revealed its position near the ostium. CBCT further confirmed the implant lodged within the left ostium, highlighting the dynamic nature of implant migration in the sinus.

Surgical approaches for implant retrieval vary depending on the location of the displaced implant and patient-specific factors [14]. Endoscopic techniques are increasingly preferred for their minimally invasive nature, superior visualization, and reduced postoperative complications [3,11]. Still, intraoral approaches, as employed in our case, can also be effective when executed with meticulous planning. By minimizing disruption to the sinus anatomy, these approaches can significantly reduce the risk of oroantral fistula formation and promote optimal healing [8,9]. The patient preferred intraoral removal under local anesthesia, as it was perceived to be less invasive; moreover, this approach provided direct access to the implant and avoided the need for general anesthesia.

The location of the incision is influenced by the position of the implant within the sinus, the condition of the sinus walls, and the surgeon’s preference [5]. The primary principle, however, is to avoid existing openings, minimize tissue damage, and reduce the risk of oroantral fistula formation [5]. Techniques such as the bone window method emphasize the importance of the incision site. Typically, the incision begins at the gingival margin and extends toward the sinus wall. This approach aims to create a bone window away from the existing implant migration site, thereby minimizing the risk of fistula development and ensuring better surgical outcomes [6,12].

Rhodium dental mirrors, known for their nonfogging and reflective properties, were essential in tracking the implant’s position within the maxillary sinus. Due to the difficulty of directly visualizing the ostium, indirect visualization via these mirrors reduced the need for excessive surgeon tilting and provided clear guidance during the procedure.

Various tools are used in removing dental implants from the maxillary sinus, adapted to the implant’s location, sinus condition, and surgical technique. Common instruments include fine suction tips for retrieving implants and clearing debris, particularly effective in hard-to-reach areas, and sinus forceps of various shapes and sizes for securing and extracting implants [7]. In this case, a custom-modified dental explorer was employed alongside traditional sinus forceps. The explorer was straightened and notched at the tip to engage the implant’s aggressive threads effectively. This tailored instrument addressed the specific challenges posed by the implant’s macrothreaded design, proving highly effective for secure removal and underscoring the value of customized surgical tools in complex cases.

The BFP is a reliable and minimally invasive option for preventing oroantral fistulas (OAF) and addressing complications following sinus surgery [5,15]. Its advantages include easy accessibility, rich vascularity, rapid healing, and low complication rates [15,16]. The adipose tissue within the BFP provides a scaffold for epithelial growth, aids epithelial migration from the gingival margin, and can also be used to reconstruct bone defects in the oral cavity. Furthermore, it is an effective method for managing complications arising from dental implants displaced into the maxillary sinus [7,16]. In the present case, the displaced dental implant was removed from the sinus, and the BFP was employed to prevent potential OAF formation. The graft not only supported mucosal closure but also minimized the risk of postoperative complications, ensuring optimal healing and functional outcomes. In this case, pseudoephedrine hydrochloride was prescribed twice daily for five days to facilitate sinus drainage and alleviate nasal congestion. Additionally, amoxicillin-clavulanate (1,000 mg twice daily for five days) was administered as a broad-spectrum antibiotic to prevent or treat potential sinus infections. Flurbiprofen was used as an analgesic to manage postoperative pain effectively. At the 10-day, one-month, and three-month follow-ups, the surgical site had healed with primary closure, and the patient remained free of complaints. Simultaneous multiple implant placements are advantageous for ensuring patient cooperation and optimizing procedural efficiency. However, whenever feasible, supplementing local anesthesia with intravenous sedation may be beneficial in minimizing patient movement and enhancing surgical precision, thereby contributing to a more controlled and predictable operative environment.

## Conclusions

This case demonstrates that retrieval of a displaced dental implant from the maxillary sinus may be successfully performed through an intraoral approach in carefully selected situations. However, as this report describes a single clinical case, the findings should be interpreted with caution. The present outcome should not be considered sufficient to support broad generalizations regarding the feasibility or superiority of this approach. Further clinical reports and larger case series are needed to better define the indications, limitations, and predictability of this management strategy.
